# Uric Acid for Cardiovascular Risk: Dr. Jekyll or Mr. Hide?

**DOI:** 10.3390/diseases4010012

**Published:** 2016-02-26

**Authors:** Cristina Vassalle, Annamaria Mazzone, Laura Sabatino, Clara Carpeggiani

**Affiliations:** Fondazione CNR-Regione Toscana G Monasterio and Istituto di Fisiologia Clinica, Italian National Research Council, Pisa I-56124, Italy; mazzone@ftgm.it (A.M.); laura.sabatino@ifc.cnr.it (L.S.); clara@ifc.cnr.it (C.C.)

**Keywords:** uric acid, health, oxidative stress, antioxidants, cardiovascular disease

## Abstract

Uric acid (UA) is a potent endogenous antioxidant. However, high concentrations of this molecule have been associated with cardiovascular disease (CVD) and renal dysfunction, involving mechanisms that include oxidative stress, inflammatory processes, and endothelial injury. Experimental and *in vitro* results suggest that this biomarker behaves like other antioxidants, which can shift from the physiological antioxidant action to a pro-oxidizing effect according to their level and to microenvironment conditions. However, data on patients (general population or CAD cohorts) are controversial, so the debate on the role of hyperuricemia as a causative factor for CVD is still ongoing. Increasing evidence indicates UA as more meaningful to assess CVD in women, even though this aspect needs deeper investigation. It will be important to identify thresholds responsible for UA “biological shift” from protective to harmful effects in different pathological conditions, and according to possible gender-related differences. In any case, UA is a low-tech and inexpensive biomarker, generally performed at patient’s hospitalization and, therefore, easily accessible information for clinicians. For these reasons, UA might represent a useful additive tool as much as a CV risk marker. Thus, in view of available evidence, progressive UA elevation with levels higher than 6 mg/dL could be considered an “alarm” for increased CV risk.

## 1. Introduction

Uric acid (UA) is a known endogenous scavenger, which provides a major part of the antioxidant capacity against oxidative and radical injury [1]. However, at high levels, UA can shift from an antioxidant to a pro-oxidant factor (shuttle capacity), depending on the characteristic of the surrounding microenvironment (e.g., UA levels, acidity, depletion of other antioxidants, reduced nitric oxide, NO, availability) [2,3]. Accordingly, high UA values have been associated with metabolic syndrome, cardiovascular disease (CVD), and renal dysfunction, involving mechanisms that favor oxidative stress, inflammation, and endothelial dysfunction [4,5]. Unlike the majority of mammals, UA is the end product of purine metabolism, as a consequence of uricase loss [6,7]. This key enzyme, which controls hyperuricemia, was lost in higher primates (humans and apes) during evolution due to gene mutations [6,7]. This adaptation may confer a survival advantage in a history epoch of food shortage and global cooling, in terms of increased life expectancy [6,7]. Moreover, the increase in UA could be a mechanism to maintain blood pressure and erect position in case of very low salt ingestion, so to benefit neuronal development and function [6,7]. The development of modern, industrial societies characterized by extreme behavioral modifications (sedentary lifestyle and unlimited food supply) has occurred in a time period too short to allow genomic and metabolic adaptation. Now, the uricase loss may represent a potential risk for health, as the prevalence of hyperuricemia is increasing in Italy and Western countries, as well as in developing countries characterized by dietary and lifestyle changes [8,9,10,11]. As an example of these effects, in the 1970s a progressive increase of mean UA values from 3.5 mg/dL in the 1920s to 6–6.5 mg/dL has been observed in the USA [12].

For its anti- and pro-oxidant effects, hyperuricemia has been associated with CVD, and its functional role is still controversial. Interestingly, recent data indicate a stronger association between UA and CVD in women than in men. The aim of the present paper is to summarize dietary sources and metabolism of UA, and to discuss available evidence on the role of UA in CVD.

## 2. Uric Acid: Dietary Source

Serum UA concentration reflects the interaction of purine intake with diet, endogenous purine metabolism, altered UA excretion (reduced glomerular filtration, or tubular secretion or increased tubular resorption) and intestinal degradation [13,14].

Food intake may significantly affect the development of hyperuricemia. A high intake of red meat and seafood, sugar sweetened drinks, and alcohol increase the risk of hyperuricemia [15]. Most of these associations are related to the content of fructose in these aliments, which is also highly present in many processed foods and snacks [16]. Alcohol may lead to hyperuricemia by increasing adenine nucleotide degradation and blood lactic acid levels, also increasing concentrations and urinary excretion of hypoxanthine and xanthine via the acceleration of adenine nucleotide degradation [17].

Although many fruits and vegetables can contain high purine levels, their natural macro- and micro- nutrients in natural ratios and proportions may likely act to optimize the biological advantages of UA natural intake with diet [18,19]. In fact, a high intake of vitamins, such as vitamin C, leads to UA reduction, probably for its uricosuric effect and competition with UA resorption through a change in the anion transportation system at proximal renal tubule level [20]. Other foods, such as low-fat dairy products, vegetables, nuts, legumes, and coffee, lowered UA by multiple mechanisms [14]. For milk-derived foods, this effect is probably related to milk-associated proteins, such as casein which has an uricosuric effect, while, for coffee, the reduction of UA seems to be attributable to other substances than caffeine, which inhibit xanthine oxidase [21,22,23].

Instead, the diffusion of (1) Western diet, with high fats and fructose content; (2) street-food (SF), rich in saturated fats and poor in fibers, vitamins, and antioxidants; and (3) sedentary habit, which characterizes our actual lifestyle, may favor hyperuricemia. In particular, two studies from Italian researchers showed evidence that SF consumption is associated directly with body mass index, larger waist circumference, higher levels of cholesterol, and UA concentrations, and inversely correlated with high-density lipoproteins cholesterol [24,25]. Moreover, high-SF consumers presented a significantly lower brachial artery flow-mediated dilatation [25].

In a recent mice experimental model, the Western diet induced cardiomyocte hypertrophy, myocardial oxidative stress, interstitial fibrosis, and impaired diastolic relaxation, and enhanced activation of the S6 kinase-1 growth pathway and the profibrotic transforming growth factor-β1/Smad2/3 signaling pathway and macrophage proinflammatory polarization [26]. Conversely, all this biomarkers improved with allopurinol treatment, which lowered cardiac xanthine oxidase, as well as serum UA levels [26]. Interestingly, some substances, such as a ginseng metabolite, compound K, a dipeptidyl peptidase-4 inhibitor, and probiotics are effective to lower UA levels in experimental models, although, currently, there is no definitive evidence and, consequently, no shared consensus on the use of such uric acid-lowering intervention to improve the outcome in the clinical CV practice [27,28,29].

The scientific community has become increasingly interested in the Mediterranean diet (MeD) and lifestyle, which includes high fruit and vegetable intake, low meat consumption, use of extra virgin oil and red wine, and daily physical activity. The main reason of this interest has to be attributed to MeD’s favorable cardiometabolic, neuronal, and beneficial health effects when compared with other dietary patterns [30,31]. The effect of MeD on UA reduction was evidenced in obese subjects more than fifteen years ago [32]. More recently, in the Attica study (2380 men and women free of cardiovascular or renal disease), the MeD score results were inversely associated with UA levels, independently from several confounders, especially in women [33]. In another general population cohort (ORISCAV-LUX, Observation of Cardiovascular Risk Factors in Luxembourg study; 1352 participants, aged 18–69 years) the MeD exhibited the best ability to induce beneficial changes in numerous biochemical markers and was significantly associated with lower levels of UA among the various dietary patterns (Recommendation Compliance Index, Diet Quality Index-International, Dietary Approaches to Stop Hypertension, and Dietary Inflammatory Index) [31]. The effect of MeD was also effective in patients with asymptomatic hyperuricemia [34]. Moreover, a higher baseline adherence to MeD is also associated with lower risk of hyperuricemia in elderly subjects at high cardiovascular risk [35]. However, other data in a small group of healthy male subjects (age 29.5 ± 5.9 years, *n* = 39) evidenced that subjects on MeD showed a statistically significant increase of UA compared either to baseline and to German diet group, which is quite surprising since the German diet is characterized by high amounts of meat and pork, sausages and butter, moderate amounts of whole-meal bread, potatoes, fruit and vegetables, dairy products, fish and eggs, moderate to high amounts of beer, sweet foods, and sugar [36].

## 3. Uric Acid, CVD, and Gender Issues

UA has been primarily identified as a powerful antioxidant; therefore, UA elevation in CVD may represent a compensatory mechanism in response to a pro-oxidative and pro-inflammatory status [37]. Nonetheless, there is growing evidence on the direct pro-oxidant properties of UA in certain circumstances of elevated concentration [38]. Accordingly, UA elevation has been associated to the reduction of NO, endothelial dysfunction, and stiffness of the arteries, hypertension, insulin resistance metabolic syndrome, and inflammation [38]. Moreover, UA levels increased with aging in both sexes [39,40]. Futhermore, UA has been related with the induction of platelet adhesiveness, smooth muscle cell proliferation, and association with coronary artery calcium levels [41,42,43]. To underline the duality of this biomarker, recent meta-analysis reached opposite conclusions on the association between UA and CV events [44,45]. In a first case (402,997 subjects), the analysis evidenced a significant association between UA and incidence of coronary heart disease, and mortality risk [44]. By contrast, in another meta-analysis (9458 incident CVD cases and 155,084 controls) the association between UA and coronary heart disease loses its significance after adjustment for other parameters in the multivariate analysis [45]. A third meta-analysis (eleven studies, 172,123 subjects) reported a significant, although modest, effect of UA, which significantly increases the risk of all-cause mortality among men (RR 1.23; 95% CI 1.08–1.42), but not women (RR 1.05; 95% CI 0.79–1.39), while risk of cardiovascular mortality appeared to be more pronounced among women (RR 1.35; 95% CI 1.06–1.72) [46]. A possible explanation of such controversial data is that adjustment for creatinine or the glomerular filtration rate is not always included in the analysis of some studies, although serum UA concentration closely depended on renal function. Another critical aspect is the definition of the threshold to indicate hyperuricemia, which becomes critical since UA retains double antioxidant and pro-oxidant capacity. In fact, among studies, there is a great heterogeneity in the threshold chosen to define hyperuricemia (from 5 to 7 mg/dL) [38]. This fact is highlighted in the most recent meta-analysis conducted on the general population, where authors found a stronger association in women, and emphasized that the CV risk markedly increases raising the UA threshold by 6 to 7 mg/dL in female subjects [47].

A possible difference in gender-related UA effects is intriguing, because although UA concentration is physiologically lower in women than in men, it is emerging to be more related with CVD in women than men [48,49,50]. Higher levels of UA in men than in women at all ages have been attributed to the role of steroids in uric acid regulation, also called “uricosuric effect”, and to the possible urate-depressing effect of estrogens in women [51]. It is known that estrogens play a cardio-protective role in women who have an overall lower cardiovascular risk than men [52]. Thus, UA elevation in women after menopause could likely represent an important hallmark of lack from estrogen protection [53,54]. Interestingly, oxidative stress results as a more powerful predictor of CAD in women than in men and, consequently, it may represent a biochemical basis for the observed gender related epidemiologic differences in CAD [55,56].

Moreover, in view of the known association between hyperuricemia and insulin resistance and diabetes, the gender-specific difference in the association of UA with CVD risk may be compatible with the stronger relationship between UA and diabetes in women, given that diabetes confers a greater risk for CVD in women than in men [38]. Accordingly, we observed a stronger association between UA and diabetes in CAD females than in males, and a significant correlation between UA levels and fasting glucose and glycated hemoglobin only in women [40]. Nonetheless, although UA is more related with hard events (overall mortality and nonfatal myocardial infarction) in CAD female patients (*n* = 843, UA cut off corresponding to 5.7 mg/dL; *n* = 2177, UA cut off 7 mg/dL for males), it does not seem to be an independent risk factors for hard events in both sexes [40]. Conversely, very recent data from two cohorts (general population of 15,083 participants in the Scottish Heart Health Extended Cohort Study, and in 13,273 CAD patients) confirmed a stronger association of UA and total mortality in female subjects than in men [57,58]. This aspect is particularly important because, although gender issues are still under evaluated in the clinical practice, fundamental biological differences and peculiarities exist between men and women, and UA could represent an interesting candidate as CV risk factor in women [59].

## 4. UA Role as CVD Maker (Mr. Hide) or Marker (Dr. Jekyll)

**Figure 1:**
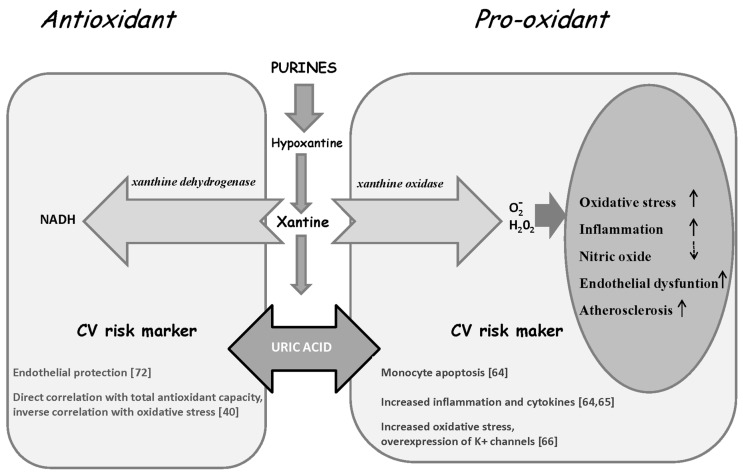
Duality of UA, which can shift from the protective antioxidant capacity to detrimental pro-oxidizing and pro-atherogenic effects according to concentration and the surrounding microenvironment.

Presently, whether UA represents an independent risk factor for cardiovascular events with a direct and causal role or if it is just a marker for an adverse risk profile is still conjectural [60,61]. UA is known as a potent antioxidant, proposed as an evolutionary alternative to the loss of ability in synthesizing ascorbate in higher primates, thus lowering the lipid peroxidation rate and counteracting the increased oxidative stress status [1,62]. Accordingly, UA’s protective role has been evidenced in several neurologic diseases, including multiple sclerosis and Parkinson’s disease [9]. These facts may argue in favor of a compensatory mechanism. Nonetheless, elevated UA might induce CV and renal disease, involving mechanisms characterized by oxidative stress, inflammation, and endothelial dysfunction [38,60]. Specifically, UA can act as a pro-oxidant by generating free radicals during its degradation through xantine oxidase activity (Figure 1). Moreover, UA results correlated with different inflammatory parameters in a general population of the InCHIANTI study and in CAD patients, although it is not clear whether UA could represent a marker of pro-inflammatory state rather than a cause of inflammation [40,63]. In this context, *in vitro* data suggest that UA induces monocyte apoptosis through the activation of both death receptor and mitochondrial-mediated pathways, and stimulates mononuclear cells to produce TNFα [64]. Moreover, recent data evidenced a role for UA in NLRP3 inflammasome activation, which is critical for the release of inflammatory cytokines [65].

In addition, a new mechanism for urate-induced CVD has been proposed [66]. In particular, treatment with urate at 7 mg/dL for 24 h in cultured mouse atrial myocytes (HL-1 cells) induced an increase in reactive oxygen species, which was abolished by the antioxidant N-acetylcysteine, and the NADPH-oxidase inhibitor apocynin [66]. Moreover, authors observed increased Kv1.5 protein expression, mediated by the NADPH oxidase-dependent oxidative stress and the ERK pathway, and suggested a role for hyperuricemia in the overexpression of K+ channels [66].

Many other mechanisms may account for the atherogenic role of UA, although a clear causal relationship between UA and CVD has never been confirmed. UA may stimulate vascular smooth cell proliferation, platelet hyper-reactivity and cyclooxigenase-2 activity, and immunity response, whereas it reduces NO availability [67]. In particular, UA (200 and 300 micromol) induced vascular smooth muscle cell proliferation and oxidative stress through the stimulation of the vascular renin-angiotensin system (increased angiotensinogen messenger RNA expression and intracellular concentrations of angiotensin II), and this effect appeared mediated by the mitogen-activated protein kinase pathway [68].

Interestingly, it has been hypothesized that circulating UA exerts protective effects on endothelial cells from oxidative stress, whereas its entry into the cell favors oxidative stress and reduces NO, evidencing the “shuttle” capacity of UA [67]. In this context, electrochemical measurement of NO release in acetylcholine-stimulated human umbilical endothelial cells (HUVECs) demonstrated that UA markedly decreased NO [69]. This result was further confirmed by organ bath experiments on mouse aortic segments [69]. Underlying mechanisms for such effects included increased arginase activity, increased intracellular superoxide formation, and reduced endothelial NO synthase phosphorylation [69]. Interestingly, UA effect on the NO pathway, which is in turn inducible by estrogen, might represent a critical “devil” triangle for the CV risk profile in women [70].

However, other data confirmed UA protective roles, in view of its antioxidant capacity. In fact, UA intravenous infusion (1000 mg) improves endothelial dysfunction in type 1 diabetes and smokers [71]. Interestingly, a recent observation added new evidence of UA as antioxidant acting to protect the endothelial function, as patients with renal hypouricemia (<2.5 mg/dL due to SLC22A12/URAT1 loss-of-function mutations) present a marked reduction of flow-mediated dilation [72]. Accordingly, we recently witnessed that UA did not represent a significant risk factor for hard events in a large population of patients referred for coronary angiography, and that UA levels correlated directly with indexes of the total antioxidant capacity (FRAP) and inversely with the oxidant counterpart (d-ROMs), thus suggesting compensatory elevation in order to prevent further damage rather than a causative role for CVD [40].

In any case, the identification of UA as a CVD culprit may be useful because UA pharmacological reduction is possible, but clearly effective only in identifying hyperuricemia as a cause rather than a consequence of clinical factors related to CVD and adverse prognosis. There are recent preliminary indications of cardiovascular and renal effects of UA lowering [73,74,75]. Interestingly, a meta-analysis focused on studies which evaluate xanthine oxidase inhibition (XOI) on clinical or surrogate markers of CVD, found that XOI improves endothelial function and levels of oxidative stress biomarkers in patients with CVD, or at risk of CVD [76]. However, only randomized controlled studies aimed at lowering UA concentration may ultimately answer this question, and they should include female patients, which might potentially benefit more from these interventions.

UA is a low-tech and inexpensive biomarker which is generally performed at a patient’s hospitalization, therefore being easily-accessible information for clinicians. Moreover, UA provides repeatable results, without presenting significant diurnal variations, although meals may affect its concentration, with significant postprandial effects. For this reason, fasting before a withdrawal is always preferable [77,78,79]. Therefore, UA might be helpful in the assessment of CV risk even without a causal role, as a risk marker.

## 5. Conclusions

Available data on the role of hyperuricemia as CV risk factor are not definitive (Table 1). However, the controversy regarding UA as a causative CV factor can be considered of low priority, as UA could represent a useful additive tool to evaluate CV risk, relevant even as a risk marker. A more critical aspect will be to define the threshold responsible for UA “biological shift” from protective to dangerous actions and the possible role of elevated UA in the interaction with other toxic substrates and reactive oxygen species in order to contribute to vessel damage and dysfunction.

Increasing evidence indicates UA as a more meaningful marker to assess CV risk in women. This fact is particularly relevant due to the lack of appropriate biomarkers to assess female CV risk, and the easy availability of this biomarker in clinical practice.

In any case, progressive elevations of UA and levels higher than 6 mg/dL should be considered an “alarm” and should induce the clinician to activate a global CV risk reduction program (including healthy dietary habits) to improve outcomes, especially in female subjects.

## Figures and Tables

**Table 1:** Hyperuricemia as risk factor for CVD: results from meta-analysis studies.

Patient Number	Subjects	Study Number	Endpoint	Odds Ratio (95%CI)	Comment	Ref.
402.997	general population	13	Coronary heart disease, CHD	1.09 (1.03–1.16)	Higher risk for CHD mortality in women	[44]
		9	CHD mortality	1.16 (1.01–1.30)		
			CHD mortality	1.12 (1.05–1.19)		
9.458	CHD patients	16	CHD	1.13 (1.07–1.20)	1.02 (CI, 0.91–1.14) in 8 studies with more complete adjustment	[45]
155.084	controls					
172.123	general population	11	Cardiovascular mortality	1.37 (1.19–1.57)	Higher risk for CV mortality in women	[46]
			all-cause mortality	1.24 (1.09–1.42)	Higher risk for all-cause mortality in men	
457.915	general population	12	CHD incidence	1.21 (1.07–1.36)	Higher risk for CHD incidence and mortality in women	[47]
237.433		7	CHD mortality	1.21 (1.00–1.46)		
12.677	complicated myocardial infarction (MI) or	3	CV mortality	1.47 (1.17–1.83)		[80]
	heart failure (HF)		all-cause mortality	1.36 (1.11–1.67)		
			HF hospitalization	1.28 (1.14–1.43)		
427.917	general population and CHD pts	5	HF incidence	1.19 (1.17–1.21)		[81]
51.552	HF patients	28	all-cause mortality	1.04 (1.02–1.06)		
			death or cardiac events	1.28 (0.97–1.70)		
